# The Automatic Proportionator Estimator Is Highly Efficient for Estimation of Total Number of Sparse Cell Populations

**DOI:** 10.3389/fnana.2018.00019

**Published:** 2018-03-21

**Authors:** Rogely W. Boyce, Hans J. G. Gundersen

**Affiliations:** ^1^Amgen Inc., Comparative Biology and Safety Sciences, Thousand Oaks, CA, United States; ^2^Aarhus University, Aarhus, Denmark

**Keywords:** proportionator, nonuniform sampling, cell number, image analysis, fractionator

## Abstract

Estimation of total number of a population of cells that are sparsely distributed in an organ or anatomically-defined region of interest represents a challenge for conventional stereological methods. In these situations, classic fractionator approaches that rely on systematic uniform random sampling are highly inefficient and, in many cases, impractical due to the intense sampling of the organ and tissue sections that is required to obtain sufficient counts for an acceptable level of precision. The proportionator, an estimator based on non-uniform sampling theory, marries automated image analysis with stereological principles and is the only estimator that provides a highly efficient and precise method to address these challenging quantification problems. In this paper, the practical considerations of the proportionator estimator and its implementation with Proportionator™ software and digital slide imaging are reviewed. The power of the proportionator as a stereological tool is illustrated in its application to the estimation of the total number of a very rare (~50/vertebrae) and sparsely distributed population of osteoprogenitor cells in mouse vertebral body. The proportionator offers a solution to neuroscientists interested in quantifying total cell number of sparse cell populations in the central and peripheral nervous system where systematic uniform random sampling-based stereological estimators are impractical.

## Introduction

The optical and physical fractionators have been the stereological method-of-choice for obtaining unbiased estimates of total cell number for nearly three decades (Gundersen, 1986; West et al., 1991). The statistical robustness of the fractionator principle relies on the precision and efficiency of systematic uniform sampling to obtain an estimate of cell number from a final fraction of the organ/region of interest for cell counting (Gundersen, 1986). The true total population number is inferred in a statistical sense from the subsample which requires the total cell population be of sufficient size such that subsampling can be performed. When the total population of the cell of interest is small and/or sparsely distributed, fractionator sampling becomes laborious and prohibitively inefficient. To obtain sufficient counts for an acceptable level of estimate precision (~100–200 counts), intense sampling of the organ and tissue sections is required because many sampled fields will not contain the cell of interest.

Gardi (Gardi et al., 2008) introduced the proportionator estimator, a unique application of non-uniform sampling based on automated image analysis-derived features combined with stereological principles. This estimator is the only estimator that provides a solution for estimating sparse cell populations, where a large fraction of the fields of view are devoid of the cell of interest at the magnification used for counting. A sparse population may be a small number of cells in a small organ or may constitute a large number in a large organ but sparsely distributed. The gain in efficiency and precision compared with systematic uniform random sampling (SURS) sampling such as classical fractionators was detailed by Gardi (Gardi et al., 2008) and later by Keller (Keller et al., 2013). The basic sampling unit of the proportionator is the tile: an area of a size and shape and position defined by the unbiased sampling frame. All tiles (or a known fraction when the total cell population is large and/or not sparsely distributed) are assigned a “weight” using automated image analysis. Weight is most commonly the area of the tile occupied by a special or immunohistochemical stain that identifies the cells of interest. The non-uniform sampling intrinsic to the proportionator avoids sampling tiles with low cell number or weight. Although the sampling may appear biased, the probability of sampling is known and is proportional to the weight, and the estimation is therefore unbiased. The tiles are then arranged in a smooth fractionator (Gundersen, 2002) according to weight which reduces variance, and then subsampled for analysis and counting. Total cell number can then be derived with a few straightforward mathematical formulas (Gardi et al., 2008).

The implementation of the proportionator in the laboratory has been facilitated by the development of Proportionator™ software (Visiopharm, Hørsholm, DK) in combination with digital slide imaging, collectively termed the automatic proportionator estimator. In this paper, practical considerations for implementation of the automatic proportionator estimator are reviewed. To illustrate the power of the automatic proportionator estimator as a stereological tool, estimation of total number of a very rare and sparsely distributed population of osteoprogenitor cells in mouse vertebrae where total number ordinarily is below 50 cells/vertebrae was performed. For in-depth presentation of the theory of the proportionator estimator, the reader is referred to additional references (Gardi et al., 2008; Gundersen et al., 2013).

## Automatic proportionator estimator: practical considerations for implementation and theory

### Section preparation and staining

Because the cell population is sparsely distributed, the volume of tissue available for analysis must be maximized. For small organs which can be routinely processed intact in paraffin, a known fraction of the organ is collected during exhaustive sectioning using fractionator sampling. Shrinkage is not a concern because the entire organ is processed in paraffin before any sampling occurs; the total number of cells is available for sampling regardless of deformation caused by shrinkage. These sections are collected as serial section pairs at each sampling interval for counting in physical disectors. Collecting disector pairs on a single slide should be done if possible to reduce the number of slides for subsequent digital scanning. For large organs, subsampling will need to be performed, and if paraffin processing is planned, special sampling designs have to be used to deal with shrinkage of subsamples during paraffin processing. The reader is referred to Gundersen et al. (2013), where numerous sampling designs are presented that account for paraffin processing for large organs where subsampling is required.

For preparation of disector sections (i.e., section pairs separated by a known distance) from paraffin blocks, thermal deformation of paraffin must be avoided. Chilling or “icing” of the block face during sectioning will result in thermal deformation and inconsistent section thickness and hence disector height. In addition, overstretching in the water bath should be avoided as this confounds alignment of the disector section pairs and the matched tile at high magnification used for counting by the software (Autodisector™, Visiopharm, described below). It is recommended sections are first placed on a room temperature water bath to collect section pairs, then transferred on uncharged slides to a warm water bath to briefly allow sections to relax, then picked up on charged slides. Section preparation is discussed in more detail in Gundersen et al. (2013).

Because some type of chromogenic staining will typically be used as the image analysis feature for weighting of the tiles, staining protocols must be optimized. Nonspecific staining or stain trapping must be avoided as the image analysis algorithm will capture staining artifacts and assign a large weight where cell count will not be proportional; this “high weight/low count” increases the variance of the estimate i.e., it decreases estimator precision.

### Details of fractionator sampling of the sections of the organ

True to its name, the fractionator is the uniform sampling of a *fixed, constant fraction* of any series of items (Gundersen, 1986) including but not restricted to that of serial sections. The rational sampling of fractionator sections is performed in a few steps using as an example, an organ of dimensions approximately 3 × 3 × 3 mm with a sparse cell population of interest:

1. Measure the height (*H* ~ 3.0 *mm* ~ 3000 μ*m*) of the organ perpendicular to the sectioning plane.
2. Decide upon a total number of section pairs *ns* in the fractionator sample. Although *ns* ~ 10 section pairs are a typically sufficient sample for obtaining acceptable precision, for a sparse cell population which may also be very inhomogeneous, an *ns* ~ 15 − 20 is suggested for the pilot study.
3. Decide upon the section thickness *t*. To avoid several practical problems (and bias) in the disector counting of cells, *t* should be thinner than the smallest particle size if the matrix is not transparent. However, in most histologic preparations, the matrix is transparent. For efficiency reasons, section thickness should approximate ¼ the height of the particle of interest. In most cases, this would typically be 3 μm. However, for a sparse population with no overprojection problems, i.e., cells are not closely packed and not superimposed in a thicker section, we selected 6 μm for a disector height. Serial sectioning the organ at 6 μm is expected to provide $\frac{H}{t}\sim \frac{3000}{6}\sim 500$ sections.
4. To obtain a sample of *ns* ~ 20 from 500 sections we need to sample every $\frac{500}{ns}\;=\;\frac{500}{20}\;=\;25$th section and collect the consecutive section to make a serial section pair, i.e., the sampling interval *si* must be fixed at precisely *si* = 25.
5. Most importantly, the sampling interval *si*, which determines the sampling fraction *sf*, is a known and fixed constant $sf\;=\;\frac{1}{si}\;=\;\frac{1}{25}$, hence the name of the fractionator.
6. Before cutting, the starting point for sampling in the first period of length 25 must be determined. The first section of the first section pair to be sampled must be taken at a random point, *R*, in the period: 1 ≤ *R* ≤ 25; the random number *R* is looked up in a random number table; a new random number is used for each block. After the first section pair, all further section pairs are sampled 25 sections apart.

When the total number of cells, $N\left(\frac{cell}{sample}\right)$, is determined in all sampled sections (using the proportionator, discussed below) the estimator of the total number per organ is simply

(1) $$N\left(cell\right):=\frac{1}{sf}\;*\;N\left(\frac{cell}{sample}\right)\;=\;25\;*\;N(\frac{cell}{sample})$$

If the average cross section of the organ has area *A* ~10,000,000 μ*m*^2^ ~10*mm*^2^, the total tissue volume to be investigated is A^*^ns^*^2^*^t ~ 2, 400, 000, 000μ*m*^3^ (about 2 cubic mm; counting both ways in the disectors is expressed in the factor of 2, described below). The sampled sections constitute $sf\;=\;\frac{1}{25}$ of the total organ and contains $\frac{1}{25}$ of the total number of sparse particles (and $\frac{1}{25}$ of the total quantity of anything else in the organ).

### Cell counting using the disector

As indicated by its name, the disector, (Sterio, 1984) is two adjacent sections separated by a known distance. On one section, an unbiased sampling and counting frame is superposed; the other section is a look-up section. A cell profile sampled according to the unbiased counting rule as illustrated is looked for in the other section. If the cell is also detected in the look-up section it is not counted. Cells sampled in the frame and not detectable in the look-up section are counted; the count of such real cells is denoted *Q*^−^(*cell*) to emphasize the negative criterion for counting.

The disector counting rule means it is particle tops that are counted: count 1 if the top is *in* the disector. It is most efficient to count in both directions of the disector: having completed the counting in section 1 (with section 2 as a look-up) use section 2 as the counting section and section 1 as the look-up section (now counting bottoms of other cells).

For the sake of unbiasedness, one should use the smallest and most contrasting cell feature as the counting unit: the nucleolus in the cell types that have strictly one per cell; in most cell types the nucleus is an optimal choice. The counting of polynucleated cells requires very special counting rules that are discussed in more detail in Gundersen et al. (2013).

Counting cells at high magnification in physical disectors has been greatly facilitated by development of the Autodisector™ software. The software provides for alignment of counting and lookup fields of view (FOV) in disector sections at high magnification used for cell counting. This is most efficient when analyses are conducted on whole slide digital images. Many digital slide scanners are compatible with the Visiopharm platform.

### The practical set-up of proportionator sampling

The above set of fractionator sections with a section sampling fraction of $\frac{1}{25}$ can now be analyzed and subsampled with the proportionator. This can be performed using either a microscope under complete computer control or digital slide images in conjunction with the Proportionator™ software. The example below outlines the general procedure using digital slide images for cell counting.

1. Sections are scanned at high magnification (“40 × objective”) on a digital slide scanner and imported into the Visiopharm software platform.
2. An image analysis algorithm is created to identify the cells of interest (typically a histochemical or immunohistochemical stain) on high resolution digital image at the magnification the “weighting” of tiles will be done for proportionator sampling.
3. In the Autodisector ™ software, superimages (lower resolution images) of the sections are created for alignment and linking of the sections. If there is a specific region of interest in the sections to which sampling will be restricted, these can be drawn. Note: The Proportionator™ combines all sampling sections or ROIs into one “supersection” of a combined area of *SumA* = ns^*^A = 200,000,000 μ*m*^2^. This is the total area which is sampled for estimating $N\left(\frac{cell}{sample}\right)$, the total number of cells in the supersection, which is $\frac{1}{25}$ of the organ.
4. Proportionator sampling is selected. The size of the unbiased sampling frame *a(fra)* which defines the tile e.g., 200 by 200 μm = 40,000 μ*m*^2^ is specified; largest frame possible is typically best. The number of tiles or sample size (22–30 typically) for each independent sampling performed with the proportionator (3 are performed, see below) is also specified.
5. The Proportionator™ applies the image analysis algorithm to the original high resolution digital slide images and assigns a weight to all tiles. The Proportionator™ software sorts these tiles by increasing then decreasing weight modeling a smooth and symmetrical distribution (known as the smooth fractionator (Gundersen, 2002), described further below).
6. The Proportionator™ performs the 3 independent samplings of tiles from the smooth fractionator but presents for examination and counting the total sample of tiles summed for the 3 samplings (e.g., 22 per sampling = 66 tiles are presented for counting). In the end, the tripartition of the complete sample enables the precision of the estimator to be estimated directly and unbiasedly discussed below.

### Proportionator sampling and determination of the sampling probability of individual tile

The Proportionator™ automatically scans all possible tiles across the high resolution digital images of all sampled fractionator sections. In the example, the supersection contains *SumA/a(fra)* ~200,000,000/40,000 ~5,000 tiles. For each tile, the proportionator automatically records the number of pixels *z*_*i*_ of the specific color.

This is the pivotal step in the proportionator sampling/estimator. The crucial information (presence of specific pixels in tiles) is sampled automatically in all 5,000 tiles. The user only has to examine for example 66 tiles but the information in all 5,000 tiles participated in their selection.

In cases of an exorbitant number of tiles when the section area is large, it is possible to sample a fraction of these, in the final estimation one just takes the tile sampling fraction in consideration. For a sparse population, the sampled number of tiles should not be below 10,000.

**Figure 1:**
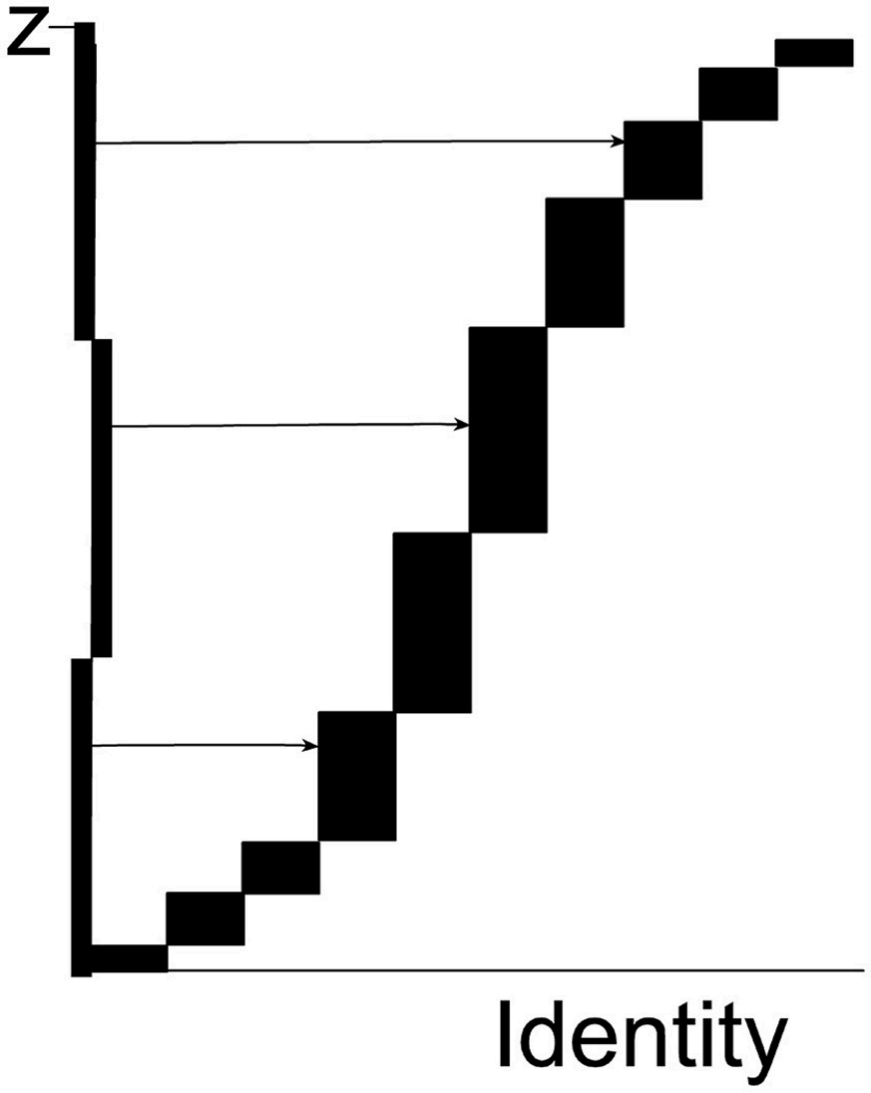
Each rectangle represents one tile. The height of each rectangle is the number *z*_*i*_of pixels of the specified color in the tile in question; the horizontal axis contains information about the location of the tile in the tessellation of all tile in the supersection. The total height of the abscissa is the total number of all specified pixels in the supersection, $\underset{5,000}{\sum }z_{i}\;=Z$ (the figure just shows 10 tiles). The ordinate is divided into *n* = 22 equidistant intervals (only 3 are shown in the figure as horizontal lines). The length of each interval on the ordinate is *T*_*z*_ = Z/22. A set of 22 horizontal and equidistant lines is drawn, each originating on its own interval (the set of lines is a systematic uniformly random sample on the ordinate). Each line intersects the vertical side of exactly one rectangle and thereby samples that rectangle. Tiles with a relatively large number of pixels have a higher sampling probability, i.e., proportionator sampling among all tiles is non-uniform. There can be hundreds or thousands of tiles with zero specific pixels (e.g., specific immunoreactivity) and they would never be sampled.

For automatic sampling, the 5,000 tiles, each with a known content of specific pixels, are arranged using the smooth fractionator (Gundersen, 2002) in a co-ordinate system in one (long!) staggered column, cf. Figure 1 which shows the arrangement for just 10 tiles. From the 5,000 tiles, a non-uniform sample of size *n* = 22 for example is drawn 3 times independently and proportional to the pixel content (i.e., weight) and the 66 tiles are presented for cell counting by the expert user.

Each rectangle, i.e., each *Tile*_*i*_, is sampled with probability $p_{i}\;=\;\frac{z_{i}}{T_{z}}$. The probability is exact: *z*_*i*_ is the exact number of special pixel in *Tile*_*i*_ and *T*_*z*_ is a large, known constant: $\underset{5,000}{\sum }z_{i}$, the sum of all the individual pixel values. As an example, let *z*_*i*_ = 17 pixel and *T*_*z*_ = 343 pixel, then ${\;p}_{i}=\frac{17}{343}=0.0496.\;$This is the probability that this particular tile was sampled; other tiles are sampled with different probabilities. It is a major advantage of the proportionator that the (many) tiles with no pixels are never sampled for counting because tiles are sampled with a probability proportional to number of special pixels contained in a tile.

From the above example, the probability *p*_*i*_ = 0.0496 is the sampling probability of this particular tile (the i'th *Tile*_*i*_) but it is also the sampling probability of any cells in the tile (*x*_*i*_ in *Tile*_*i*_). Therefore, when a count of *x*_*i*_ = 2 cells was observed in the disector for this particular tile, that event had probability *p*_*i*_ = 0.0496.

### The estimation of the total number of cells, N(cell), in the complete organ

There exists a mathematical theorem, the *Horvitz-Thompson theorem*, which states that the contribution of a count from a tile to total cell number in the supersection is directly proportional to the count divided by the probability of sampling the tile based on weight. This is mathematically defined for the particular tile with a count of 2 and probability of sampling based on weight as 0.0496:

(2) $$X_{i}\;=\;\frac{x_{i}}{p_{i}}\;=\;\frac{2}{0.0496}\;=\;40.3$$

where *X*_*i*_ is the contribution from the count of *x*_*i*_ = 2 to the total number of cells in the supersection. The computation is performed automatically by the Proportionator™ software. That is, by counting 2 cells in a volume of tissue defined by the area of one random tile with a known probability and the disector height, the contribution of 40.3 from this sample to the total supersection can be computed. By analogy, if a cell is sampled in the tile with probability $\frac{1}{21}$ there must be 21 such cells in the supersection–on average.

This process is repeated for all 22 tiles in one subsample of tiles which then provides the first estimate of the total number of cells $\underset{22}{\sum }X_{i}\;=\;$ *X*1 = 224, for example, in the supersection. Two further repetitions of independent samplings for the remaining 44 tiles provide *X*2 = 124 and *X*3 = 214, respectively. Three independent samplings are recommended to achieve a stable CE.

The $mean\left(X\right)\;=\;\frac{X1+X2+X3}{3}\;=\;188$ divided by the fractionator section sampling fraction, $sf=\frac{1}{25}$, is the final estimate of the global total number of cells

(3) $$N\left(cell\right)\frac{mean\left(X\right)}{2*sf}\;=\;\frac{188}{2*\frac{1}{25}}\;=\;2,950$$

The factor of 2 compensates for counting both directions in the disector because this doubles the volume in which cells are counted. This is the result of the study of one animal.

### The precision of the estimator and the variability of animals in a group

The differences between *X*1, *X*2, *X*3, estimates from 3 independent samplings of the supersection are indicative of the imprecision of the estimator of total number of cells. If very similar, the precision must be good, and vice versa. In fact, the imprecision of the estimator is simply defined:

(4) $$CE\left(N\right):=\frac{SEM\left(X1,X2,X3\right)}{mean\left(X\right)}\;=\;\frac{31.8}{188}\;=\;0.17$$

This is an extraordinarily simple equation and unbiased estimator of precision of the measurement. This simple mathematical expression for unbiased estimation of precision, *CE* (coefficient of error), is unique to the proportionator.

At the end of the pilot phase (e.g., 3–6 animals per group), compute the average imprecision of the estimator:

(5) $$\bar{CE_{est}}\left(N\right):\;=\;\sqrt{\left(\sum_{4}CE_{i}^{2}\right)/4}\sim 0.13$$

This is the mean CE for a group of 4 animals.

Compute also

(6) $$CV_{obs}\left(N\right):=\;\frac{SD\left(est1,\;est2,\;est3,est4\right)}{mean\;estimate}\sim 0.32$$

which is the observed, computed variability among the estimates from four pilot animal; *SD* is the ordinary standard deviation. The numbers in Equations (5, 6) are arbitrary numbers used as examples.

Note that one cannot overestimate the value of a pilot study which provides very valuable information about the precision of the estimation procedure, $\bar{CE_{est}}\left(N\right)$, and the biological variability of the test animals, *CV*_*obs*_(*N*). No optimization of the main (large) study design is possible without the pilot study, see below.

## Example of application of the automated proportionator: pilot study for estimation of total number of osteoprogenitor cells in mouse vertebrae

### Material and methods

#### Animals

C57BL/6 male mice (6–7 weeks old) carrying the SOX9-creERt and dT-tomato reporter, were used in this pilot study. Lineage tracing studies have demonstrated that early mesenchymal progenitors defined by promoter activity of Sox9 and subsequent expression of tomato protein differentiate into chondrocytes, osteoblasts, stromal cells and adipocytes during endochondral bone development (Ono et al., 2014). Mice were administered subcutaneously either vehicle (*n* = 3) or 50 mg/kg sclerostin antibody (*n* = 3) (r13c7, supplied by Amgen Inc.) on Day 1. On Day 6, mice were administered tamoxifen (2 mg intraperitoneally) and then terminated by cervical dislocation under isoflurane anesthesia on Day 10. Thoracic vertebrae T11-13 were collected, cleaned of soft tissue and fixed in 4% paraformaldehyde (PFA) for 2–3 days at 4°C. Vertebral samples used in this pilot study were graciously provided by Drs. Deepak Balani and Henry Kronenberg, Massachusetts General Hospital, Boston MA. Mice were group-housed in sterile, ventilated microisolator cages on corn cob bedding in a facility accredited by the Association for Assessment and Accreditation of Laboratory Animal Care. All procedures were conducted in compliance with the Guide for the Care and Use of Laboratory Animals approved by Massachusetts General Hospital's Institutional Animal Care and Use Committee. Animals were provided *ad libitum* access to pelleted feed (LabDiet 5010) and water (Standard drinking water of Boston, MA; pH 7.8) via Hydropac. Animals were maintained on a 12-h light/12-h dark cycle in rooms at 64° to 79°F with 30–70% humidity under pathogen-free conditions.

Vertebral segments consisting of 3 thoracic vertebrae/animal were decalcified in 10% EDTA + 2% PFA, then routinely processed in paraffin. Blocks were exhaustively sectioned at 6 μm and using SURS section pairs were collected every 36 micrometers using an automated microtome calibrated to the section thickness (*ssf* = 1/6). Six micron thick sections were chosen for the pilot because the target cell population was expected to be sparse based on qualitative evaluation of sections stained for tomato protein. Number of section pairs ranged from 13 to 18 per animal. Section pairs were mounted on charged slides and immunohistochemically stained for tomato protein using a rabbit polyclonal antibody to Red Fluorescent Protein at 1:500 (Abcam, #ab62341) on a Ventana Discovery Ultra™ (Ventana, Tuscon AZ), an automated immunostaining system. Briefly, sections were incubated with primary antibody for 1 h, followed by anti-rabbit HQ (Ventana, reference no. 760-4815) for 12 min and anti-HQ horseradish peroxidase (Ventana, reference no. 760-4820) for 12 min, developed with diaminobenzadine, then counterstained with hematoxylin.

#### Stereological methods

**Figure 2:**
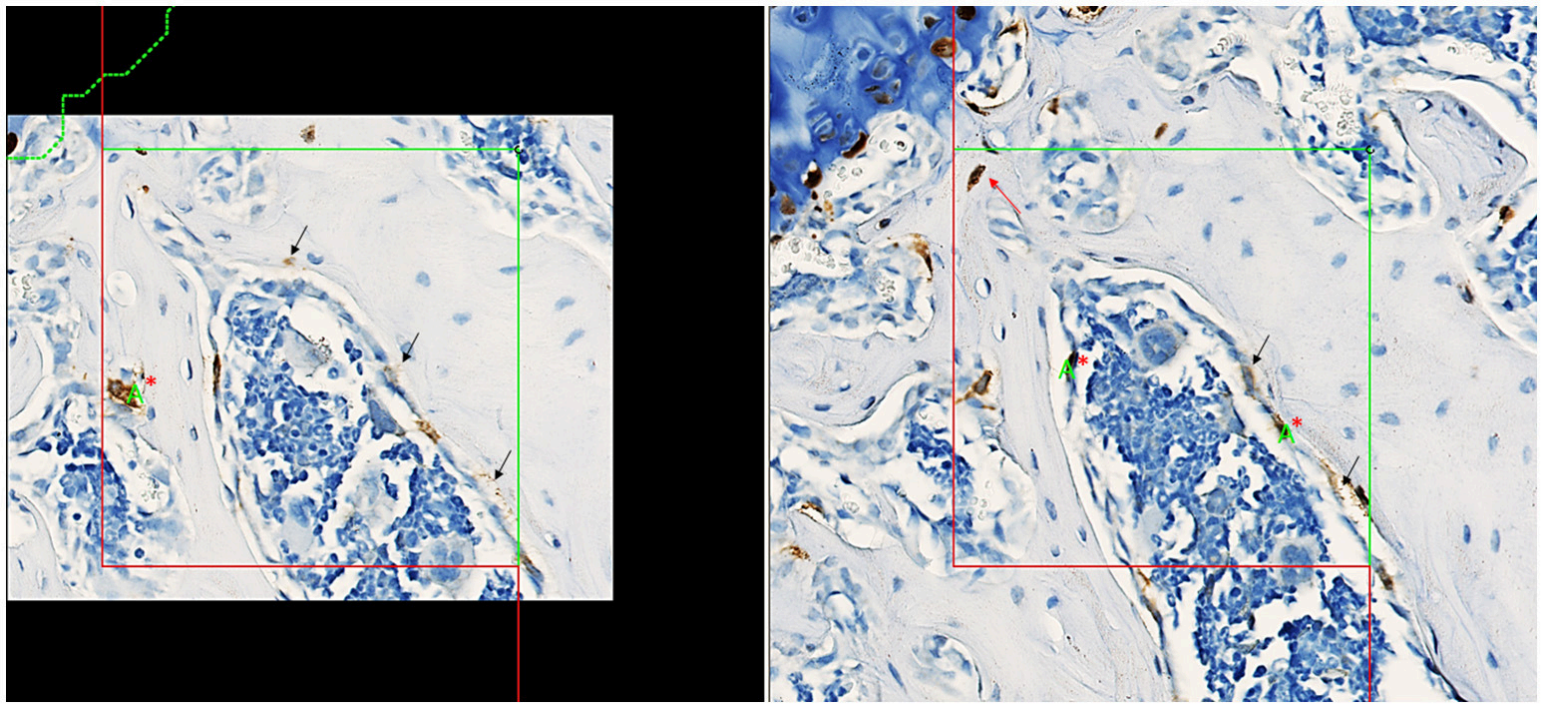
Screenshots captured from the Visiopharm platform of matched fields of view in a disector pair with aligned unbiased counting frames, counting section on left, look-up section on right. Nuclei present in tomato-positive osteoprogenitor cells (brown cytoplasm) in the counting section are counted if it is not present in the look-up section. Counting in the other direction of the disector, nuclei present in tomato-positive osteoprogenitor cells in the look-up section are counted if not present in the counting section. The final total count is divided by 2 to correct for counting in both directions. Counting in both directions increases the count and improves precision of the estimate. The green letter “A” with red “^*^” denotes a “count”; a total count of 3 was recorded for this tile counting in both directions of the disector. Tomato-positive osteocytes (red arrow) were rare events and were not included in the counts. Staining interpreted as non-specific is denoted by black arrows.

Stained slides were scanned at 40X objective magnification using the Hamamatsu Nanozoomer™ whole slide scanner and imported into the AutoDisector™; superimages were then created by the software. Superimages of disector pairs were linked and aligned, and the region of interest (ROI) was defined. A ROI was drawn around each vertebral body (excluding the cortical bone and growth plate); 3 vertebral bodies were used in analysis for each animal to increase the total tissue volume. An image analysis algorithm was created to identify tomato-positive cells and stored to guide Proportionator™ sampling. Three independent samplings of 60 tiles each were performed for a total of 180 tiles at the “40X magnification” setting. An unbiased counting frame (200 × 200 μm) was applied to define the proportionator tile and the number of tomato-positive osteoprogenitor cells within the bone marrow was counted, which included tomato-positive cells on the bone surface interpreted to be osteoblasts. Occasional tomato-positive cells were observed within the bone matrix (consistent with osteocytes); these cells were not included in the analysis. The nucleus was used as the unique counting feature. Counting was performed on both directions of the disector (Figure 2).

The total number of tomato-positive osteoprogenitor cells for each independent sampling was determined by the following calculation: $\frac{\sum X}{2}*\frac{1}{ssf}$, where ∑ *X* is the sum of the weighted counts (divided by 2 to account for counting in both directions of the disector) and *ssf* is the section sampling fraction, which was 1/6. The mean of the 3 independent samplings was calculated for each animal and was divided by the 3 (number of vertebrae used in analysis) to determine the number of tomato-positive cells per vertebra for each animal. CE was calculated per Equation (4). The mean, standard deviation, CE, and coefficient of variation (CV) for each group was calculated.

### Results

The results of the pilot study are sumarized in Table 1. Total tomato-positive osteoprogenitor counts across 3 vertebral bodies/animal ranged from 19 to 80 and total number/vertebrae ranged from 27 to 187. Sclerostin antibody increased the mean number by approximately 3-fold but with a high CV (~60%). Mean CE of the proportionator estimator in both groups was ~9–10%.

**Table 1:** Summary of stereological data of tomato-positive osteoprogenitors (OP) labeled with tomato protein in mouse vertebrae.

	**Vehicle**	**Sclerostin antibody**
**INDIVIDUAL TOTAL COUNT SUMMED FOR 3 SAMPLING**		
	36	33
	19	48
	28	80
Mean total count/group	28	54
**INDIVIDUAL ESTIMATE OF TOTAL NUMBER OF OP CELLS/VERTEBRAE**		
	41	90
	38	58
	27	187
Mean total number of OP cells/group	35	112
SD	7.4	67.2
CV	0.21	0.60
**INDIVIDUAL CE**		
	0.05	0.11
	0.08	0.14
	0.12	0.01
Mean CE/group	0.088	0.103

### Optimizing the relationship of the data quality (precision) to the necessary effort

At the end of the pilot study it is possible to get an answer to the crucial question: is the stereological estimator precise enough for the purpose of the study?

$\bar{CE_{est}}$*(N)*, the imprecision of the estimator, is under full control of the investigator: the larger the sample the smaller the $\bar{CE_{est}}$*(N)*. “Sample” can be any or all of (1) the fractionator sections, (2) the area of the counting frame, and (3) the number of tiles sampled. The design outlined above results in a certain precision of the estimator in the organ under study, $\bar{CE_{est}}\left(N\right)\sim 0.088$ in the vehicle group and 0.103 in the treatment group. The question is, is that low enough for the purpose of the study.

The variability among animal estimates, C*V*_*obs*_(*N*), and the estimator imprecision, $\bar{CE_{est}}$*(N)*, are dependent in a very useful way:

(7) $$CV_{obs}^{2}\left(N\right)\;=\;CV_{ani}^{2}\left(N\right)+\bar{CE_{est}^{2}}\left(N\right)$$

which states that the estimator imprecision, $\bar{CE_{est}^{2}}\left(N\right)$, inflates the real (unknown) variability between animals (the biological variation), $CV_{ani}^{2}\left(N\right)$, thus contributing to the observed $CV_{obs}^{2}{\left(N\right)}_{.}$ If two groups show very different $CV_{obs}^{2}\left(N\right)$ but have similar $\bar{CE_{est}^{2}}\left(N\right)$ then their biological variation must be very different, cf. the example in Table 1.

Clearly, $\bar{CE_{est}^{2}}\left(N\right)$ should be small compared to $CV_{obs}^{2}\left(N\right)$, cf. Equation (7). The question is how small? The simple answer is provided by the general inequality applicable to each group of the study (and very useful for all stereological estimators) is estimation of the Precision Range of an Optimally Balanced Estimator (PROBE) (Gundersen et al., 2013):

(8) $$PROBE:2<\frac{CV_{obs}^{2}\left(N\right)}{\bar{CE_{est}^{2}}\left(N\right)}<4$$

The above inequalities leads to 1 of 3 possible conclusions:

PROBE larger than 4: the precision of the estimator may be too good for the purpose. If convenient, reduce the workload where it is heaviest.
PROBE between 2 and 4: The precision is adequate for the purpose.
PROBE below 2. The case where the estimator imprecision is too large for the purpose. The question is which part of the estimator should be studied more intensively.

1. If the organ is inhomogeneous at the scale of sections, increase the number of sections (e.g., from 20 to 30–40 in each organ).
2. If the sections are very inhomogeneous at the scale of tile or the total count is too small for the purpose: increase the frame size to its maximum and increase the number of sampled tiles (e.g., from 22^*^3 to 35^*^3).

Substituting the values from Equations (5, 6), the PROBE ratio $\frac{CV_{obs}^{2}\left(N\right)}{\bar{CE_{est}^{2}}\left(N\right)}$ in Equation (8) becomes

(9) $$\frac{CV_{obs}^{2}\left(N\right)}{\bar{CE_{est}^{2}}\left(N\right)}\;=\;5.7$$

for the vehicle group and

(10) $$\frac{CV_{obs}^{2}\left(N\right)}{\bar{CE_{est}^{2}}\left(N\right)}\;=\;34$$

for the sclerostin antibody group.

Since 5.7 is larger than 4, cf. Equation (8), we may optimize the sampling effort in the main study, probably by moderately reducing the number of fractionator sections from an average of 15 to 12: there is no indication of a pronounced inhomogeneity among the sections, they look mostly the same. Also, the number of tiles sampled may be reduced from 60 ^*^3 to 40 ^*^ 3.

The value of 34 is much larger than 4 and one might think of reducing the sampling for sclerostin antibody group by a large measure. However, under ordinary scientific paradigms one must study groups of animals blindly, i.e., one can only use one sampling protocol for the entire study. That just emphasizes the importance of the pilot study and the subsequent optimization of the study estimator.

There are no set rules for performing the optimization, except to use caution and change the sample sizes by a proportion less than that of the PROBE number in relation to the PROBE limit. As an example, the PROBE number of the vehicle group is 5.7 which is roughly a factor 3 larger than the PROBE limit of 2.0. Consequently, we have reduced the sample sizes by a factor less than 3.

Due to the low number of observations in the pilot study the PROBE values are not very precise and one should use common sense in the interpretation. If some of the values are unexpected or counter intuitive, it is worth considering another pilot study.

Note that all of the above pertains to sparse population of cell. For the pilot study of ordinary organs with many cells, it is recommended that 10 fractionator sections and 15 tiles with 3 independent samplings (total 45 tiles for counting) are used for the pilot study.

### The hopeless case of the fractionator estimator of total number in sparse populations

What would the imprecision be for the good-old, no-nonsense fractionator estimator of total number using uniform sampling provide in this sparse osteoprogenitor population?

One answer is the *CE*_*fract est*_(*N*) when, in the presented example, all 1,900 tiles or unbiased counting frames in all ~15 sampled sections are studied (the tile sampling fraction is therefore 1.00). The total number of cells in all sampled sections is 35, which is also the fractionator total count. To reach a count of 1 cell, it is necessary to study $\frac{1900}{35}\sim 54$ empty tiles. The imprecision of the fractionator estimator is

$$CE_{fract\;est}\left(N\right)\;=\;\frac{1}{\sqrt{count}}\;=\;\frac{1}{\sqrt{35}}\sim 0.169.$$

Even when studying all 1,900 tiles the fractionator has a PROBE value of $\frac{CV_{obs}^{2}\left(N\right)}{\bar{CE_{est}^{2}}\left(N\right)}\;=\;1.5$, well below the lower limit of 2.0 for acceptable imprecision. In truth, a Herculean effort with a poor outcome.

## Discussion

Estimation of the total number of osteoprogenitors with SOX9 promoter activity in the mouse vertebral body is a nice example to illustrate why the automated proportionator is the first ever practical estimator of the total number in sparse cell populations. The automatic proportionator offers many advantages compared with other sampling and estimation strategies, notably the relative immunity of the proportionator to sparseness and inhomogeneity.

The degree of sparseness of these osteoprogenitors in the mouse vertebra has not been properly defined, but one may index this by a ratio of the total number of zero count tiles/tiles with a count (usually a count of 1). On this scale, these osteoprogenitors in the vertebra have a sparseness of 54. It is a remarkable feature of the automatic proportionator that its efficiency does not depend on the degree of sparseness. To a first approximation it is equally efficient in cell populations with the degree of sparseness ranging from 10 to 1,000. Like sparseness, inhomogeneity is difficult to define rigorously, but one may think of a large variability between sampling items with respect to cell density that may exist independently among tiles and among sections. Inhomogeneity makes the fractionator inefficient, whereas the proportionator is largely unaffected. In the raw data, inhomogeneity was evident between sections, but there was also marked inhomogeneity between tiles. Cells were preferentially located near the endplates, notably adjacent to the endocortex, which has been described for these cells using bone clearing techniques and 3-dimensional imaging of whole mouse vertebral bodies (Greenbaum et al., 2017). Inhomogeneity or non-uniform distribution of cell populations, which may or may not be sparse, is a common situation in the neurosciences where proportionator sampling can offer significant improvements in efficiency.

Another unique feature of the proportionator is the relationship between the absolute count ∑ *Q*^−^ and the weighted estimator imprecision CE (N). Under uniform sampling the imprecision of the vehicle group mean ∑ *Q*^−^ of 28 is $\frac{1}{\sqrt{28}}\;=\;0.189$. However, under weighted sampling the CE (28) is 0.088, cf. Table 1, and is computed from the differences of the three individual estimates based on three independent sampling of 60 tiles.

There are a number of practical details to address for optimal performance of the proportionator. Near perfect sections are required as loss of tissue is evidently a loss of information, i.e., a bias. Near perfect staining is required because the proportionator is particularly sensitive to nonspecific staining of the background and section edges. Moderate staining problems increase the CE, but noteworthy does not result in a bias, but uneven staining of sections leads to reduced efficiency. Bias can be introduced in cases where staining does not detect the cell of interest or staining artifact prevents identification of the cell. Tiles may be encountered with staining or sectioning artifacts that confound performing a count. Appendix details the method to address these non-useable tiles.

Another consideration in regard to efficiency of the proportionator is the amount of computing time required to perform the weighting. In the current version of the Proportionator ™ software, weighting is performed on the high resolution digital images, critically important when weighting on small stained features in a cell, such as the cytoplasm or nucleus. When the section area is small, weighting on 100% of the tiles is not burdensome, as this can be performed unattended by the user. However, when section area is large, some fraction of the tiles may be sampled; this option is currently available in the Proportionator™ software.

Although this paper focused on applications using chromogenic immunophenotyping, immunofluorescent-stained thin sections can be used with the Proportionator™ software and integrated into the automated workflow if an immunofluorescence slide scanner is available. Proportionator sampling of immunofluorescent-stained thick sections combined with the optical disector can be performed with the Visiopharm platform configured with an automated microscope and appropriate camera. Although not an aspect of the present study it is worthy of mention that the proportionator is equally efficient as a sampling and estimation protocol for all stereological modalities: total number, total length, total surface, total volume and all the particle size estimators and size distributions (Gundersen et al., 2013).

In conclusion, the automated proportionator estimator is the only practical stereological solution for obtaining estimates of total number of sparse cell populations.

## Author contributions

HJGG and RWB are responsible for the conception and design, analysis and interpretation of the work; drafting, revising and final approval of the manuscript; and accountable for accuracy and integrity of the work.

### Conflict of interest statement

RWB is a former Amgen employee and currently a consultant for Amgen through Beechy Ridge ToxPath LLC. The other author declares that the research was conducted in the absence of any commercial or financial relationships that could be construed as a potential conflict of interest.

## Appendix

### Non-useable tiles

Unavoidably in practice, technical problems may prevent a sampled tile from being correctly evaluated with respect to the objects under study; simple examples being incorrectly focusing of the image or the local loss of section substance. Since the correct estimate is based on the distinct sampling probability of the tile, *p*_*i*_, the user cannot instead select a nearby tile: that tile has a genuinely unknown sampling probability, incorporating, among other factors, the probability that the non-useable tile was indeed non-useable! As a result, no contribution to the universe total from the non-useable tile can be generated, and this unequivocally renders the estimator (negatively) biased.

A correct procedure in the face of non-useable tiles (under a critical assumption) at this stage may be as follows.

1. The total number of non-useable tiles in the sample turns out to be *n*_*n*−*u*_ = 2 of a sample total of *n* = 22. The biased estimate of the total geometric feature of interest such as total number from the incomplete procedure is *X*_*n*_*u*__, where the suffix indicates that it is based on a sample size of only *n*_*u*_ = *n*−*n*_*n*−*u*_ = 22−2 = 20 tiles.
2. Reusing the tessellation and weights of the incomplete procedure, the user specifies a second sample of precisely *n*_*n*−*u*_ = 2 tiles. The software thus resamples the complete set of tiles with a sampling period of *Z*/_*n*_*n*−*u*__ and the user provides the correct count *x*_*i*_ for each tile. The resulting estimate is *X*_*n*−*u*_. This is an unbiased estimate of the total geometric feature of interest, but evidently rather imprecise, provided *n*_*n*−*u*_ is a small number. If there happens to be non-useable tiles in this complementary sample, the whole sample is discarded and the correction procedure is repeated.
3. The original estimate, *X*_*n*_*u*__, is biased because it is short by the contribution from *n*_*n*−*u*_ = 20 tiles, which were sampled with probabilities $p_{i}=z_{i}/\left[\frac{Z}{n}\right]$. The second estimate almost fulfills the bill: it is the sum of the contributions from *n*_*n*−*u*_ = 2 tiles, but they were sampled with probabilities $\;p_{i}=z_{i}/\left[\frac{Z}{n_{n-u}}\right]$, off by just a known constant. It follows that the second estimate may be recomputed to provide the missing contribution: Δ*X*_*n*−*u*_ = (*n*_*n*−*u*_/*n*)**X*_*n*−*u*_ = (2/22)**X*_*n*−*u*_.
4. The unbiased estimate of total geometric feature of interest is *X*_*n*_*u*__ + Δ*X*_*n*−*u*_–now based on the required *n* tiles.

The correction of *X*_*n*_*u*__ is 0 if *X*_*n*−*u*_ = 0, obviously, i. e. in this case *X*_*n*_*u*__ happens to coincide with an unbiased estimate.

In the split-sample procedure, each sample of 22 observations must be corrected separately.

The assumption underlying the correction is that the non-useable tiles are a uniform sample from the total weight (only then does the correction by an extra uniform sample work). Some technical problems are plausibly independent of the objects under study, like incorrect focusing of the image. The really bad news are folds in the section. They are genuinely impossible to correct for completely (the loss of information is 3 times the loss of area) and must be avoided as far as at all possible. They are also quite likely related to the local structure of the tissue and may not be uniformly positioned. The best that can be said in such cases is that the result of the correction is likely to be less biased than the uncorrected estimate–but that is not guaranteed.
